# Population Genetics of the Asian Buffalo Leech (*Hirudinaria manillensis*) in Southern China Based on Mitochondrial Protein-Coding Genes

**DOI:** 10.3390/biology14080926

**Published:** 2025-07-23

**Authors:** Gonghua Lin, Jingjing Yin, Wenting Zhang, Zuhao Huang, Zichao Liu, Huanhuan Chen, Lizhou Tang, Fang Zhao

**Affiliations:** 1 School of Life Sciences, Key Laboratory of Jiangxi Province for Functional Biology and Pollution Control in Red Soil Regions, Jinggangshan University, Ji’an 343009, China; lingonghua@jgsu.edu.cn (G.L.); m15656142610@163.com (J.Y.); lkr48ansheng@163.com (W.Z.); hzhow@163.com (Z.H.); 2 School of Agriculture and Life Sciences, Kunming University, Kunming 650214, China; abclzc@aliyun.com; 3 College of Biological Resource and Food Engineering, Qujing Normal University, Qujing 655011, China; chhuanhuan@163.com; 4 College of Life Sciences, Jiangxi Normal University, Nanchang 330022, China

**Keywords:** *Hirudinaria manillensis*, MitPCGs, genetic diversity, genetic structure, haplotype phylogenetics, population demography

## Abstract

Leeches hold significant medical and pharmaceutical value for antithrombotic treatments, yet their genetic diversity patterns remain poorly understood. We performed population genetic analyses on seven *Hirudinaria manillensis* populations from southern China using mitochondrial protein-coding genes (MitPCGs). Complete sequences of all 13 MitPCGs were obtained from 74 individuals. Haplotype diversity exhibited a logarithmic relationship with the gene length (*R*^2^ = 0.858, *p* < 0.001), while nucleotide diversity showed a near-perfect alternating low-high pattern (*Z* = 2.938, *p* = 0.003). Concatenated sequence analyses indicated high haplotype diversity (>0.5) and low nucleotide diversity (<0.005) across all populations, suggesting a historical bottleneck followed by rapid expansion and mutation accumulation. The haplotype network, haplotype phylogenetics, and genetic structure analyses revealed moderate genetic differentiation across populations, dividing them into three clades: a basal Yunnan population (YNHH), sub-basal Guangxi populations (GXGG, GXLZ, and GXYL), and distal Guangdong/Hainan populations (GDMM, GDZJ, and HNDA). Analysis of historical population demography revealed five phases from ancient to recent times (P1–5): growth, prolonged stability, rapid decline, rapid growth, and secondary decline. These phases correlate strongly with past climatic events, demonstrating that glacial–interglacial cycles profoundly impacted the leech’s effective population size. This study provides a key scientific basis for *H. manillensis* resource conservation and utilization.

## Simple Summary

Leeches are important segmented worms valued for medical and pharmaceutical uses. In this study, we investigated the population genetics of the Asian buffalo leech (*Hirudinaria manillensis*) from southern China using mitochondrial markers. We obtained complete genetic sequences of 13 genes from 74 leeches collected at seven locations. We found that haplotype diversity goes up as the gene length increases, while nucleotide diversity displayed an alternating pattern of low and high values. High haplotype diversity coupled with low nucleotide diversity across all populations indicates a historical population bottleneck followed by rapid growth and mutation buildup. Further analysis revealed moderate genetic differentiation, resolving populations into three primary divergence clusters: the oldest (Yunnan), intermediate (Guangxi), and youngest (Guangdong and Hainan). When examining population size changes over time, we identified five phases: initial growth, prolonged stability, a sharp decline, rapid regrowth, and a subsequent decrease. These shifts correlated with historical climate changes—particularly ice ages—which significantly influenced leech population sizes. This study provides key genetic insights to help conserve and utilize *H. manillensis* resources.

## 1. Introduction

Leeches belong to the phylum Annelida, class Hirudinea. There are about 680 species of leeches in the world, and about 100 species in China [1,2]. Many species feed on mammalian blood and secrete various anticoagulant substances with significant medical and pharmaceutical applications [3]. Since the 17th century, European countries have widely employed the European medicinal leech (*Hirudo medicinalis*) in leech therapy to treat various inflammatory conditions or to prevent arterial thrombosis after surgical procedures, thereby improving surgical success rates [4]. In China, leeches are traditional medicinal agents valued for blood-breaking and stasis-dispelling properties, as well as for promoting menstruation [5]. They serve as principal components in numerous formulas for treating cardiovascular and peripheral vascular diseases [6,7].

Historically, leech research has primarily focused on the identification and application of anticoagulant components (such as hirudin, antistasin, and destabilase), with less attention paid to other biological or ecological aspects. Population genetics is a branch of genetics that studies the distribution, dynamics, and driving mechanisms of genetic variation within biological populations [8]. Geographical landscapes and climatic events stand as fundamental forces sculpting the genetic nature of animal life across evolutionary time. Vast mountain ranges, sprawling oceans, and meandering river systems, or even merely physical distance, have long acted as natural boundaries for fragmenting populations and limiting gene flow [9]. Meanwhile, climatic upheavals, from the icy pulses of Quaternary glaciations to the gradual warming of recent epochs, have driven sweeping demographic shifts [10]. Together, these geographical and climatic drivers have shaped not merely local variations but the broader patterns of genetic adaptation and biodiversity that define life’s evolutionary journey.

Conducting population genetic studies on leeches helps elucidate the characteristics and formation mechanisms of their genetic diversity, ultimately benefiting the utilization and conservation of leech genetic resources. Due to challenges such as difficult sample collection, current population genetic research on leeches remains relatively scarce. In 2012, Bielecki and Polok analyzed genetic variations in three leeches, *Erpobdella testacea*, *Glossiphonia complanata*, and *Hemiclepis marginata*, using RAPD (random amplified polymorphic DNA) assays, and found their gene diversity fell within ranges recorded in variable invertebrates [11]. In 2016, using TRAP (target region amplified polymorphism) and SSR (simple sequence repeat) markers, Liu et al. conducted analyses of genetic structure and genetic diversity on three leech species, *Hirudo nipponia*, *Poecilobdella manillensis*, and *Whitmania pigra* [12]. They discovered that these leeches exhibited high genetic diversity at the species level but low genetic diversity at the population level. Notably, these amplified fragment length polymorphism (AFLP) markers typically provide data for only a limited number of loci, making them inadequate for supporting complex population structure analyses. Furthermore, the genetic loci corresponding to these molecular markers exhibit significant species specificity, hindering comparative studies of population genetic characteristics across different species.

Mitochondria are present in the vast majority of eukaryotic cells and contain a mitochondrial genome independent of nuclear chromosomes. The animal mitochondrial genome generally comprises 13 protein-coding genes (MitPCGs) [13]. Due to their unique evolutionary characteristics, these genes serve as ideal molecular markers for population genetic studies [14]. With the development of sequencing technology, the mitochondrial genomes of various leeches have been reported [15]. However, there are few examples of using mitochondrial genes in leech population genetics research. In 2020, Yue et al. used the mitochondrial *CYTB* gene to analyze the genetic populations of *W. pigra* across Jiangsu, China. They found that this species exhibited high genetic diversity, low genetic differentiation among populations, and relatively stable historical population dynamics [16]. Recently, Popa et al. used mitochondrial *COI* as well as *12S* markers to document the distribution of Hirudo verbana in Romania. Their study revealed that this species is currently undergoing population dispersal, with wetland coverage and elevation as the primary ecological variables influencing its distribution [17].

The Asian buffalo leech (*Hirudinaria manillensis*) is the representative species of the genus *Hirudinaria* within the family Hirudinidae. It is primarily distributed across Southeast Asian countries, including China, the Philippines, Malaysia, and Vietnam, deriving its specific epithet from its type locality in the Philippines [2]. This leech feeds on the blood of mammals such as water buffaloes (*Bubalus bubalis*) and exhibits a significantly larger body size compared to common medicinal leeches (*Hirudo* spp.). Its antithrombotic activity markedly surpasses that of commonly used medicinal leech species from the genera *Hirudo* and *Whitmania* [18]. Consequently, *H. manillensis* has long been regarded as a crucial zoological source of antithrombotic agents, extensively utilized in treating cardiovascular diseases. Recent studies reveal that *H. manillensis* possesses over 70 antithrombotic-related genes, indicating substantial development potential [19]. In China, its distribution encompasses provinces including Guangdong, Guangxi, Hainan, and Fujian [2]. However, wild populations have experienced dramatic declines in recent years due to intensified harvesting and environmental degradation. Current understanding of the population genetic characteristics of *H. manillensis* remains limited, constraining research, utilization, and conservation efforts for this medicinal organism. This study conducted genome resequencing on seven *H. manillensis* populations from southern China. MitPCGs were extracted from all individuals to perform analyses of genetic diversity, haplotype variation, genetic structure, and historical population dynamics. These investigations systematically characterize the population genetics of *H. manillensis* while providing a scientific foundation for utilizing its genetic resources.

## 2. Materials and Methods

### 2.1. Sampling and Sequencing

**Figure 1:**
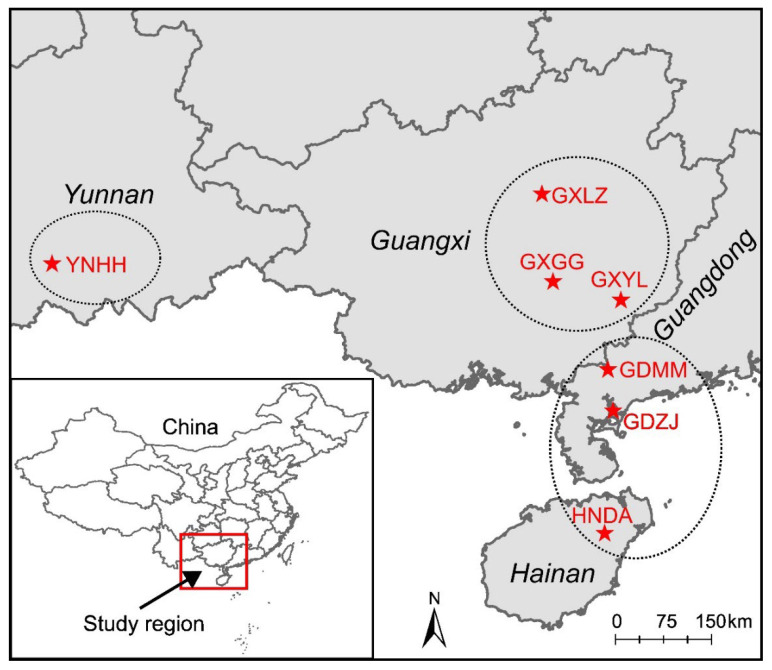
Study region and geographic distributions of the *Hirudinaria manillensis* samples (the red stars indicate the seven sampling localities).

Live specimens of *H. manillensis* were collected from seven populations across four provinces (Guangxi, Guangdong, Hainan, and Yunnan; see Table 1 and Figure 1) in June 2023. The specimens were identified morphologically according to the key table of Genus *Hirudinaria* in Fauna Sinica (Annelida Hirudinea) [2]. From each sampling site, 10–12 adult leeches were randomly selected. The anterior part of each specimen was excised, and total genomic DNA was extracted using the DNeasy Blood and Tissue Kit (QIAGEN, Düsseldorf, Germany). Qualified DNA extracts were used to construct ~350 bp libraries with Illumina-compatible reagents, followed by whole-genome resequencing (150 bp paired-end) on the BGISeq platform. Raw sequencing reads were processed with fastp v0.20.0 [20] to remove adapters and low-quality regions, generating clean reads for each sample that were subsequently used in downstream bioinformatic analyses.

### 2.2. MtPCGs Sequence Extraction

The clean reads from genome resequencing were de novo assembled using MEGAHIT v1.2.9 [21], generating contig sequence files for each sample. MtPCGs from the published *H. manillensis* mitochondrial genome (GenBank accession No. NC_023925.1) were served as bait sequences to retrieve homologous sequences from the unigenes files via BLAST v2.13.0+ [22]. Retrieved sequences were aligned using MEGA v11.0.13 [23], and homologous regions were extracted. Notably, due to high local variability in certain genes, some genomic reads failed to assemble despite being sequenced. For such cases, MIRAbait v4.9.6 [24] was used to extract homologous reads from the raw genomic data, followed by alignment and verification in MEGA.

### 2.3. Genetic Diversity Analysis

Coding sequences of each gene were merged into individual FASTA files. Multiple sequence alignment was performed using the “Align by MUSCLE” function in MEGA. DnaSP v6 [25] was employed to calculate the number of variable sites (VS), haplotype number (HN), haplotype diversity (Hd), and nucleotide diversity (Pi) for each gene. All MtPCGs were concatenated, and the same metrics were computed across different populations.

### 2.4. Network and Phylogenetic Analysis of Haplotypes

Concatenated MtPCGs were imported into HapSolutely v0.2.2 [26] to generate haplotype networks under the Fitchi model. All haplotype sequences were extracted from the concatenated using DnaSP. The MtPCGs of the closely related species *Hirudinaria javanica* were downloaded from GenBank (accession No. NC_061323.1). Using the concatenated MtPCGs of *H. javanica* as the outgroup, a maximum-likelihood phylogenetic tree was constructed with IQ-TREE v2.2.0 [27] with the best substitution model selected via ModelFinder (embedded in IQ-TREE) and 1000 bootstrap replicates. Resulting trees were visualized using FigTree v1.4.4 (http://tree.bio.ed.ac.uk/software/Figtree/, accessed on 10 April 2025) and edited in Inkscape v1.3 (https://inkscape.org, accessed on 10 April 2025).

### 2.5. Population Genetic Structure Analysis

The concatenated MtPCGs of all *H. manillensis* samples were used for structure analysis in STRUCTURE v2.3.4 [28] for K = 2 to K = 7. The parameters were the Length of the Burning Period = 5000, the Number of MCMC Reps after Burning = 50,000, and the Number of Iterations = 20. Results were summarized using structureHarvester [29], and Delta K values were calculated. Genetic structure plots were generated with the pophelper v2.3.1 R package [30]. AMOVA (Analyses of Molecular Variance) and pairwise genetic differentiations (*F_ST_* values) of the populations were calculated using Arlequin v3.5 [31]. The number of permutations was set to be 1000, and the significance level was set to 0.05.

### 2.6. Population Dynamics

Population dynamics of *H. manillensis* were inferred using the Bayesian Skyline model in BEAST v1.10.4 [32]. First, the optimal nucleotide substitution model was determined by jModelTest v2.1.10 [33]. The alignment was then loaded into BEAUti v1.10.4 [32], and the following settings were used: Substitution Model, the best fit model from jModelTest; Base frequencies, Estimated; Clock Type, Uncorrelated relaxed lognormal clock (prior: fixed). Given the absence of estimated mitochondrial evolutionary rates for annelids, we used the “standard” mitochondrial substitution rate for invertebrates (1.15% substitutions per million years) [34,35].

## 3. Results

### 3.1. Sequence Variation in Different Genes

We obtained sequences of all MtPCGs from 74 samples across seven populations. Among these, *ND5* was the longest gene (1710 bp), while *ATP8* was the shortest (153 bp). The combined length of all 13 genes was 11,040 bp, with no insertion–deletion mutations detected. All sequence data have been deposited in Supplementary File S1. Alignment revealed substantial genetic variation: a total of 318 variable sites were identified, and each gene contained 3–66 variable sites and 4–36 haplotypes. Consistently, *ND5* exhibited the highest number of variable sites and haplotypes, while *ATP8* showed the lowest, indicating a strong influence of sequence length on variation. After length normalization, *ND5* displayed the highest haplotype diversity, whereas *ATP8* had the lowest. For nucleotide diversity, *COIII* ranked highest, followed by the longest gene, *ND5*, while the shortest, *ATP8*, remained the lowest (Table 2).

**Figure 2:**
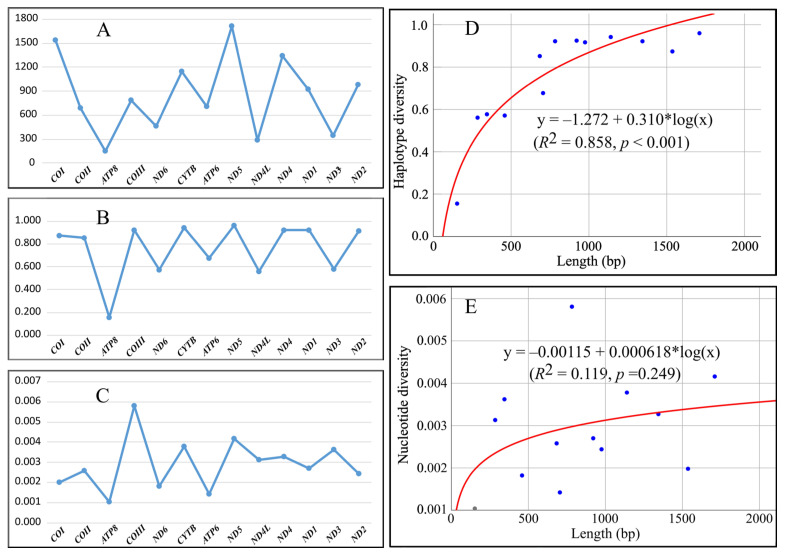
Gene length (**A**), haplotype diversity (**B**), and nucleotide diversity (**C**) of the 13 MitPCGs, and the lognormal regression of haplotype diversity (**D**) and nucleotide diversity (**E**) against gene length.

Interestingly, gene length, haplotype diversity, and nucleotide diversity exhibited periodic patterns (Figure 2A–C). After binarizing data (large values = 1, small values = 0), run tests showed near-significant (*Z* = 1.772, *p* = 0.076) deviation from randomness for gene length and haplotype diversity, while nucleotide diversity displayed highly significant deviation (*Z* = 2.938, *p* = 0.003), revealing a near-perfect “small-large-small-large” periodicity. Curve-fitting indicated that haplotype diversity followed a logarithmic relationship with the gene length (*R*^2^ = 0.858, *p* < 0.001), whereas nucleotide diversity showed a weak, non-significant correlation (*R*^2^ = 0.119, *p* > 0.05) (Figure 2D,E).

### 3.2. Population Genetic Diversity

Combining all MtPCGs, we calculated genetic diversity for the overall dataset and individual populations. A total of 61 haplotypes were identified. The overall haplotype diversity and nucleotide diversity were 0.989 and 0.00309, respectively. Population-specific analyses (Table 3) showed that all populations except YNHH had ≥ 59 variable sites, 9–10 haplotypes, Hd ≥ 0.970, and Pi ≥ 0.00180.

### 3.3. Haplotype Network Analysis

**Figure 3:**
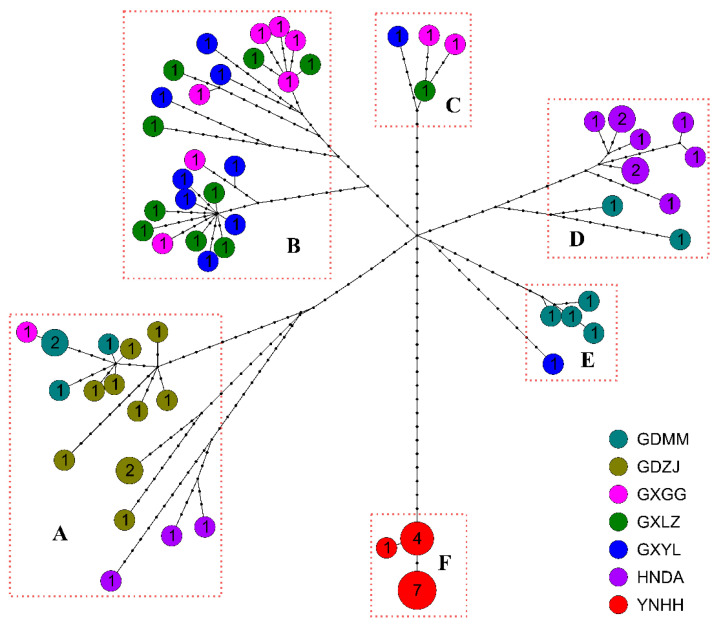
Haplotype network of the concatenated MitPCGs in *H. manillensis* samples (the number within each circle indicates the frequency of each haplotype; letters A–F denote seven distinct branches).

The haplotype network formed seven distinct branches (A–F) with a radial topology (Figure 3). Branch A predominantly comprised haplotypes from GDZJ, with partial contributions from GDMM, HNDA, and GXGG. Branch B included haplotypes from three Guangxi populations (GXYL, GXLZ, and GXGG). Branch C contained only four haplotypes but involved the same three Guangxi populations. Branch D combined GDMM and HNDA haplotypes. Branch E involved GDMM and GXYL. Branch F exclusively contained haplotypes from the YNHH population. Over 20 mutation steps were detected between each pair of branches, indicating a high degree of differentiation. Specifically, while the YNHH haplotypes were separated by more than 30 steps from those in other localities, only 1–2 mutation steps occurred within the YNHH branch (branch F).

### 3.4. Phylogenetic Analysis

**Figure 4:**
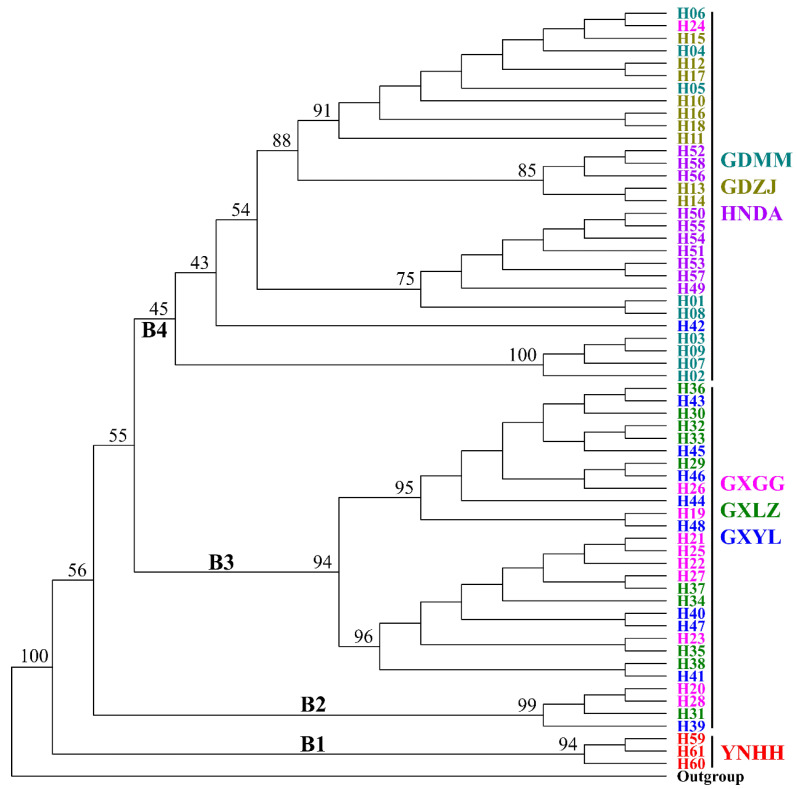
Phylogenetic relationships among the haplotypes (H01–H60) of the concatenated *H. manillensis* MitPCGs (B1–B4 denote four monophyletic clades; numbers beside each node represent percentages of bootstrap values).

Using *H. javanica* as the outgroup, phylogenetic analysis of *H. manillensis* haplotypes resolved four monophyletic clades (B1–B4). Clades B1–B3 received strong support (>90% bootstrap), while B4 had weak support (45%) (Figure 4). Clade B1 (basal position) consisted solely of three YNHH haplotypes. Clade B2 (sub-basal position) contained four haplotypes from Guangxi populations (GXYL, GXLZ, and GXGG). Sister clades B3 and B4 were recovered: B3 (similar to B2) included 24 Guangxi haplotypes (GXYL, GXLZ, and GXGG), while B4 comprised 29 haplotypes from GDMM, HNDA, and GXGG, plus one exception (haplotype H24 from GXGG).

### 3.5. Genetic Structure and Genetic Differentiation

**Figure 5:**
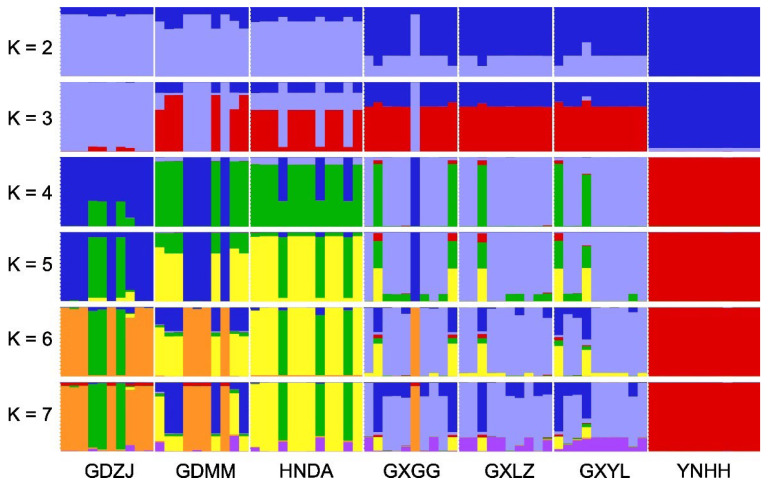
Genetic structure patterns of the *H. manillensis* populations with different clustering strategies (K = 2–7).

As shown in Figure 5, the STRUCTURE analysis revealed a turning point at K = 3 (Delta K = 2.309). Across all clustering strategies (K = 2–7), individuals from the YNHH population showed no shared genetic information with other populations, indicating distinct genetic characteristics. Meanwhile, the three populations from Guangxi Province (GXGG, GXLZ, and GXYL) consistently exhibited highly similar genetic compositions across all K-values, suggesting shared genetic backgrounds with minimal differentiation. At K = 2–3, populations GDZJ, GDMM, and HNDA displayed close genetic affinities, with GDZJ showing lower genetic complexity. At K ≥ 4, these three populations exhibited overlapping genetic features, implying limited differentiation.

AMOVA results indicated that 46.26% of the genetic variation occurred between populations, while 53.74% occurred within populations. Combined with the significant result (*p* < 0.001), this suggests moderate genetic differentiation among populations. Pairwise genetic differentiation analysis (Table 4) revealed significantly high *F_ST_* values (>0.6) between YNHH and all other populations, confirming substantial divergence. Conversely, the three Guangxi populations (GXGG, GXLZ, and GXYL) showed negligible genetic differentiation (*F_ST_* ≤ 0.010, *p* > 0.05). Populations GDZJ, GDMM, and HNDA showed significant but moderate differentiation (*F_ST_* ≤ 0.5), consistent with limited divergence. These results align closely with the phylogenetic and STRUCTURE analysis. Collectively, the seven populations form three genetic clusters: a basal cluster (YNHH), a sub-basal cluster (GXGG, GXLZ, and GXYL), and a distal cluster (GDZJ, GDMM, and HNDA).

### 3.6. Historical Population Dynamics

**Figure 6:**
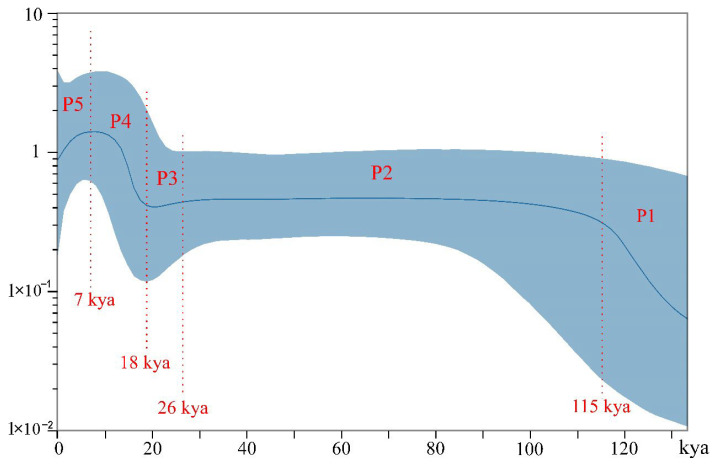
Historical population dynamics based on the Bayesian Skyline model and all *H. manillensis* samples (kya means kiloyear ago).

Model selection identified HKY+I as the optimal model for reconstructing the historical demography of *H. manillensis* using coalescent theory. Based on climatic events, population dynamics (Figure 6) were divided into five phases (P1–P5): P1 (133–115 kya, kiloyear ago), population expansion, coinciding with the Eemian Interglacial (130–115 kya) [36], suggesting warm interglacial conditions drove sustained growth. P2 (115–26.5 kya), stabilization during the early-to-mid Last Glacial Period (110–26.5 kya) [37], where persistent cooling halted population growth. P3 (26–18 kya), a decline during the Last Glacial Maximum (LGM) during 26.5–19 or 24–17 kya [38,39], with abrupt cooling causing a sharp population reduction. P4 (19–7 kya), rapid expansion post-LGM, linked to climatic warming [38,39]. P5 (7 kya–present), rapid decline associated with gradual global cooling and intensified anthropogenic impacts after 7 kya [40].

## 4. Discussion

This study extracted all MtPCGs from 74 *H. manillensis* individuals across seven populations using genome resequencing and assembly, followed by population genetic analyses. Analysis of overall genetic variation revealed significant differences in variation levels among genes. Interestingly, both haplotype diversity and nucleotide diversity exhibited periodic fluctuations. Further investigation showed that the lengths of these 13 genes also followed periodic changes, with haplotype diversity demonstrating an almost perfect logarithmic increase relative to gene length, indicating gene length as a key determinant of haplotype diversity.

Haplotype diversity is defined as the probability that two randomly sampled sequences differ [41]. Theoretically, longer sequences increase the probability of divergence between two randomly selected sequences. Thus, the strong positive correlation between haplotype diversity and gene length aligns with expectations. Conversely, nucleotide diversity is defined as the average number of nucleotide differences per site in pairwise sequence comparisons [41], a metric inherently independent of sequence length. Consequently, the weak correlation between nucleotide diversity and gene length suggests uneven evolutionary rates among genes. Strikingly, the nucleotide diversity values of the 13 MtPCGs exhibited a near-perfect periodic pattern of alternating low and high values—a phenomenon unreported previously, warranting further investigation into its mechanism.

Analysis based on concatenated MtPCGs revealed haplotype diversity > 0.5 and nucleotide diversity < 0.005 across all populations. According to Grant and Bowen [42], this pattern indicates a “population bottleneck followed by rapid population growth and accumulation of mutations.” This aligns with historical demographic analyses, which confirm that *H. manillensis* experienced a short-term population decline during the Last Glacial Maximum, followed by rapid expansion. Our historical population dynamic reconstruction further details distinct phases: population growth (P1), prolonged stability (P2), rapid decline (P3), rapid growth (P4), and secondary decline (P5)—all closely correlated with historical climatic events. As blood-feeding organisms, their survival depends critically on host abundance. Glacial–interglacial cycles profoundly impacted faunal dynamics, inevitably influencing the leech’s effective population size [43]. This highlights blood-feeding animals as valuable models for studying species-environment historical interactions.

Haplotype network analysis revealed seven star-like haplogroups. While most comprised individuals from multiple populations, Group F consisted exclusively of YNHH specimens from Yunnan Province. Crucially, >30 mutation steps separated YNHH haplotypes from all other populations, whereas only 1–2 mutation steps occurred within this group, indicating marked genetic isolation in this population. Genetic structure analysis further indicated that YNHH was genetically distinct, three Guangxi populations (GXGG, GXLZ, and GXYL) were inseparable, and the remaining three (GDZJ, GDMM, and HNDA) showed minor differentiation. Integrating with haplotype network, phylogenetic, and genetic structure results, these populations could be placed into three groups: the earliest diverging Yunnan population (YNHH), intermediate Guangxi populations (GXGG, GXLZ, and GXYL), and most recent Guangdong/Hainan populations (GDMM, GDZJ, and HNDA) (Figure 1).

It should be noted that some nodes in the haplotype-based phylogenetic tree exhibited weak support (<75% bootstrap values, a common threshold in genetic studies). Additionally, AMOVA revealed that under half of the genetic variation occurred between populations. Together, these results indicate moderate genetic differentiation among H. manillensis populations, which appears directly influenced by geographical factors. For example, the isolation of the YNHH population is expected given its distant location, while haplotypes from the three Guangxi populations (GXGG, GXLZ, and GXYL) were closely related, likely due to their geographic proximity. Surprisingly, however, HNDA (Hainan Island) exhibited low genetic differentiation from mainland populations (GDZJ and GDMM). As freshwater organisms, *H. manillensis* cannot traverse the 20-km-wide Qiongzhou Strait [44] via swimming or marine fish parasitism. However, paleoclimate studies indicate a 130–150 m sea-level drop during the LGM [45], transforming the strait into land [46]. We propose that leeches dispersed to Hainan during this period. If confirmed, this would indicate that climatic events not only shaped historical population dynamics but also influenced genetic structure of *H. manillensis*.

As a crucial antithrombotic medicinal resource, geographic authenticity is a key quality indicator. Previous research revealed significant divergence in hirudin sequences and expression levels among populations [47]. Clarifying phylogenetic relationships is thus essential for authenticity evaluation. Haplotype-based phylogeny placed the YNHH population at the basal position, followed by divergence of Guangxi populations and later southern Guangdong/Hainan populations. Future work will compare antithrombotic protein activities across these regions to establish scientific authenticity criteria. Yunnan Province, China’s richest *Hirudinaria* diversity hotspot, harbors three previously reported species: *H. javanica*, *H. similis*, and *H. yunnanensis* [48]. Our study reports the first wild *H. manillensis* population in Yunnan, reinforcing this region’s status as a *Hirudinaria* diversity center. Given the basal position of YNHH, we hypothesize Yunnan as the origin of *H. manillensis*. Notably, YNHH showed lower genetic diversity and pronounced differentiation from other populations, warranting priority in conservation genetics efforts.

As noted above, *H. manillensis* is distributed across multiple Asian countries. However, due to the difficulty of sampling wild populations, this study was restricted to seven locations in southern China. The limited sample size and geographical scope may have resulted in an underestimation of the species’ genetic diversity. Future studies will expand sampling across a broader geographic range to better assess both its genetic diversity and population structure.

## 5. Conclusions

This study reveals significant genetic diversity patterns among different MitPCGs and among populations of *H. manillensis* across southern China. The haplotype diversity showed a logarithmic pattern with gene length, while nucleotide diversity displayed an alternating pattern of low and high values. The high haplotype diversity coupled with low nucleotide diversity indicates a historical population bottleneck followed by rapid expansion and mutation accumulation. Phylogenetic and genetic structure analyses delineate three distinct population groups: the ancient Yunnan lineage, intermediate Guangxi populations, and the more recently diverged Guangdong/Hainan group. Crucially, historical demographic reconstructions identify five population phases (growth, stability, decline, rapid growth, and later decline) that align closely with past climatic oscillations, particularly ice age cycles. These findings demonstrate that paleoclimate dynamics profoundly shaped the species’ population size and structure. This research provides essential genetic insights for the informed conservation and sustainable utilization of this medicinally valuable leech species.

## Figures and Tables

**Table 1:** Basic information of *Hirudinaria manillensis* samplings.

Locality	City, Province	Longitude	Latitude	Sample Size
GDMM	Maoming, Guangdong	110.393	21.829	10
GDZJ	Zhanjiang, Guangdong	110.463	21.250	10
GXGG	Guigang, Guangxi	109.620	23.065	10
GXLZ	Liuzhou, Guangxi	109.465	24.300	10
GXYL	Yulin, Guangxi	110.574	22.805	10
HNDA	Dingan, Hainan	110.348	19.525	12
YNHH	Honghe, Yunnan	102.580	23.315	12

**Table 2:** Genetic variation in each mitochondrial protein-coding gene (MitPCG) of all *H. manillensis* samples.

Gene	Length	VS	HN	Hd ± SD	Pi ± SD
*COI*	1536	34	34	0.874 ± 0.029	0.00198 ± 0.00015
*COII*	684	17	19	0.852 ± 0.029	0.00258 ± 0.00023
*ATP8*	153	3	4	0.155 ± 0.056	0.00104 ± 0.00039
*COIII*	783	33	25	0.922 ± 0.014	0.00581 ± 0.00024
*ND6*	459	14	12	0.571 ± 0.067	0.00182 ± 0.00031
*CYTB*	1140	35	31	0.942 ± 0.013	0.00378 ± 0.00021
*ATP6*	705	13	15	0.677 ± 0.054	0.00142 ± 0.00018
*ND5*	1710	66	36	0.960 ± 0.010	0.00416 ± 0.00023
*ND4L*	285	9	9	0.561 ± 0.064	0.00313 ± 0.00064
*ND4*	1344	41	29	0.922 ± 0.017	0.00327 ± 0.00015
*ND1*	921	20	23	0.925 ± 0.013	0.00270 ± 0.00012
*ND3*	345	8	7	0.577 ± 0.058	0.00362 ± 0.00051
*ND2*	975	25	24	0.917 ± 0.015	0.00244 ± 0.00015

Note: VS, number of variable sites; HN, number of haplotypes; Hd, haplotype diversity; Pi, nucleotide diversity.

**Table 3:** Genetic variation in the concatenated MitPCGs in each *H. manillensis* population.

Population	VS	HN	Haplotypes	Hd ± SD	Pi ± SD
GDMM	68	9	H01–H09	0.978 ± 0.054	0.00245 ± 0.00026
GDZJ	59	9	H10–H18	0.978 ± 0.054	0.00180 ± 0.00035
GXGG	98	10	H19–H28	1.000 ± 0.045	0.00236 ± 0.00040
GXLZ	71	10	H29–H38	1.000 ± 0.045	0.00183 ± 0.00026
GXYL	88	10	H39–H48	1.000 ± 0.045	0.00214 ± 0.00030
HNDA	75	10	H49–H58	0.970 ± 0.044	0.00200 ± 0.00047
YNHH	3	3	H59–H61	0.591 ± 0.108	0.00011 ± 0.00002
Total	318	61	—	0.989 ± 0.006	0.00309 ± 0.00008

**Table 4:** Pairwise genetic differentiation (*F_ST_*) of the *H. manillensis* populations.

Population	GDMM	GDZJ	GXGG	GXLZ	GXYL	HNDA	YNHH
GDMM	—	0.008	0.009	<0.001	<0.001	<0.001	<0.001
GDZJ	0.189	—	<0.001	<0.001	<0.001	<0.001	<0.001
GXGG	0.215	0.411	—	0.309	0.345	<0.001	<0.001
GXLZ	0.342	0.528	0.010	—	0.980	<0.001	<0.001
GXYL	0.298	0.490	0.009	0.000	—	<0.001	<0.001
HNDA	0.279	0.445	0.351	0.435	0.395	—	<0.001
YNHH	0.675	0.792	0.667	0.738	0.700	0.725	—

Note: the dash (—) denotes omitted comparisons between identical populations, the below diagonal indicates the pairwise *F_ST_*, and the upper diagonal indicates the *p* values of the differentiations.

## Data Availability

Data are contained within the article and Supplementary Files.

## Supplementary Materials

The following supporting information can be downloaded at https://www.mdpi.com/article/10.3390/biology14080926/s1, File S1: all MitPCGs of the 74 *H. manillensis* samples.

## Abbreviations

MitPCGs	mitochondrial protein-coding genes
VS	number of variable sites
HN	number of haplotypes
Hd	haplotype diversity
Pi	nucleotide diversity
SD	standard deviation
